# Changes in ventilation distribution in children with neuromuscular disease using the insufflator/exsufflator technique: an observational study

**DOI:** 10.1038/s41598-022-11190-z

**Published:** 2022-04-29

**Authors:** Carmen Casaulta, Florence Messerli, Romy Rodriguez, Andrea Klein, Thomas Riedel

**Affiliations:** 1 Division of Paediatric Pulmonology, Department of Paediatrics, Inselspital, Bern University Hospital, University of Bern, Freiburgstrasse 15, 3010 Bern, Switzerland; 2 Division of Paediatric Neurology, Department of Paediatrics, Inselspital, Bern University Hospital, Univeristy of Bern, Freiburgstrasse 15, 3010 Bern, Switzerland; 3 Paediatric Neurology, University Childrens Hospital Basel, UKBB, University of Basel, Spitalstrasse 33, 4056 Basel, Switzerland; 4 Division of Paediatric Intensive Care, Department of Paediatrics, Inselspital, Bern University Hospital, Univeristy of Bern, Freiburgstrasse 15, 3010 Bern, Switzerland; 5 Department of Paediatrics, Cantonal Hospital Graubuenden, Loestrasse 170, 7000 Chur, Switzerland

**Keywords:** Neuromuscular disease, Paediatric research

## Abstract

Patients with neuromuscular disease often suffer from weak and ineffective cough resulting in mucus retention and increased risk for chest infections. Different airway clearance techniques have been proposed, one of them being the insufflator/exsufflator technique. So far, the immediate physiological effects of the insufflator/exsufflator technique on ventilation distribution and lung volumes are not known. We aimed to describe the immediate effects of the insufflator/exsufflator technique on different lung volumes, forced flows and ventilation distribution. Eight subjects (age 5.8–15.2 years) performed lung function tests including spirometry, multiple breath washout and electrical impedance tomography before and after a regular a chest physiotherapy session with an insufflator/exsufflator device. Forced lung volumes and flows as well as parameters of ventilation distribution derived from multiple breath washout and electrical impedance tomography were compared to assess the short-term effect of the therapy. In this small group of stable paediatric subjects with neuromuscular disease we could not demonstrate any short-term effects of insufflation/exsufflation manoeuvres on lung volumes, expiratory flows and ventilation distribution. With the currently used protocol of the insufflation/exsufflation manoeuvre, we cannot demonstrate any immediate changes in lung function.

## Background

Effective cough is a protective mechanism against respiratory tract infections and an important part of mucus clearance of the lung. Ineffective cough is a major cause of morbidity and mortality in patients with neuromuscular disease (NMD). Inspiratory and expiratory muscle weakness, hypoventilation and increasingly impaired cough competence are thought to cause frequent atelectasis and chest infections^1,2^. Additionally, the progressive decline in lung volume is associated with ventilation inhomogeneity^3^. A normal cough requires the inspiratory muscles to inspire to up to 85–90% of vital capacity (VC) followed by rapid closure of the glottis. Contraction of the expiratory muscles (abdominal and intercostal) causes an intrapleural pressure of 190 cmH2O and more which, in combination with the opening of the glottis, generates transient peak cough flows (PCF) of 360–1200 l/min^4,5^. While normally the inspiratory volume reaches 80–90% of VC before coughing, for an effective cough at least 50% of VC must be inspired^6,7^. Due to muscle weakness and reduced respiratory system compliance neither the deep inspiration nor the forced expiratory manoeuvre is feasible for patients with NMD. This reduction in compliance is most probably due to micro-atelectasis caused by inspiratory muscle weakness and also due to changes in the elastic recoil of the lung and reduced chest wall compliance^8–10^.

Chest physiotherapy is an important cornerstone of the regular daily treatment in these patients. Depending on cooperation different techniques are used ranging from completely passive vibration vests to active breathing techniques^11–13^. All these techniques are time and energy consuming. During respiratory infections patients are quickly exhausted and prone to the vicious cycle of secretions, fatigue and further accumulation of secretions.

Cough assist techniques were developed for patients who present with weak cough. The therapy basically pursues two goals. First, it increases the expiratory airflow that occurs during cough, by assisting inspiration and/or expiration, thus increasing cough efficacy. Second, it aims to maintain lung volume and respiratory system compliance by active insufflation of air. A mechanical insufflator/exsufflator uses positive pressure via a facemask or mouthpiece to deliver a maximal lung inspiratory volume, followed by an abrupt switch to negative pressure to the airway for forced expiration, which should transport mucus to the central airways and out of the patient. The rapid change from positive to negative pressure simulates airflow changes experienced during a normal cough manoeuver with the high expiratory airflow carrying mucus upstream. Mellies and Goebel demonstrated that the pressure required to achieve the highest PCF in young adults with NMD was 27mbar^14^. Daily use of the insufflator/exsufflator device leads to a reduction in acute pulmonary exacerbations, better clearing during the exacerbations and better overall ventilation while preventing atelectasis and hypoxia^3,15–17^.

Already back in 1952 Barach and Beck demonstrated clinical and radiographic improvement in 92 out of 103 acutely ill patients with respiratory tract infections with the use of a cough assist device, in a study that included 72 patients with lung disease and 27 with skeletal or neuromuscular disease^18^. Greater improvements were seen in patients with NMD. A similar effectiveness in airway clearance was seen with conventional physiotherapy but the treatment time needed to clear secretions was longer with the latter treatment^15^.

The safety, tolerance and clinical effectiveness of the mechanical insufflation/exsufflation in 62 paediatric patients with NMD was studied by Miske et al.^16^ the authors concluded that the use of a cough assist device was safe, well tolerated and effective in preventing pulmonary complications in 90% of their population. A retrospective study by Veldhoen et al.^17^ revealed a significantly lower number of respiratory tract infections requiring hospital admission after the introduction of the mechanical insufflation/exsufflation. Stehling et al.^3^ could demonstrate an increase in vital capacity within one year after the introduction of the therapy and its stabilisation in the second year. In the mentioned studies, no barotrauma such as air leaks have been reported, although there are some case reports in adult patients^19,20^.

Despite the low level of evidence for long-term benefits the British Thoracic Society published recommendations for the use of augmented cough techniques in children with ineffective cough^21^.

Having only few studies on long-term effects, there is even less literature on short-term effects of mechanical insufflation/exsufflation on lung function parameters, especially in children. In a group of stable subjects with Duchenne muscular dystrophy (DMD) including some adolescents, a single treatment showed a change in breathing pattern, i.e. decreased breathing rate and less shallow breathing. No changes in lung-volume recruitment or unassisted cough peak flow could be demonstrated^22^. Another study in a small group of adult subjects with DMD demonstrated a short-lasting positive effect on chest-wall motion asymmetry and a small but significant increase of vital capacity^23^.We therefore aimed to quantitatively and qualitatively describe the short-term effect of the insufflation/exsufflation manoeuvres on ventilation distribution and other lung function parameters in subjects with NMD. We hypothesised that an insufflation/exsufflation treatment session leads to short-term changes in ventilation inhomogeneity.

## Material and methods

Patients with NMD in stable conditions using a mechanical insufflator/exsufflator regularly at home were recruited at the specialised NMD outpatient clinics of a tertiary center, the University Children’s Hospital of Bern, after written informed consent by the parents. Patients were screened consecutively at each visit by the involved physiotherapist.

This observational study was approved by the Ethics committee of the Canton of Bern, Switzerland (KEK Nr. 2018-01507) and performed according to the current version of the Declaration of Helsinki and the Swiss Law for Human Research. The study was funded through a grant from the Swiss Foundation for Research on Muscle Diseases. (FSRMM; www.fsrmm.ch) The foundation was not involved in the planning or conduct of the experiments, data analysis and interpretation, the writing of the manuscript or its submission.

### Inclusion criteria

- confirmed diagnosis of a NMD
- functional status: non-ambulatory
- age > 5 years and < 16 years
- able to cooperate
- daily home-use of cough assist as reported by the caregivers

### Exclusion criteria

- acute respiratory infection (marked increase in cough, change in sputum amount or colour, fever or malaise)
- oxygen dependency (defined as need for oxygen to achieve a transcutaneous oxygen saturation of ≥ 92%)
- skin lesions at the chest

### Observed standardised procedure

After the baseline assessment, the participants performed their usual insufflator/exsufflator therapy session under supervision of a physiotherapist. Commonly in our institution a therapy session consists of 5 series of 5 insufflation/exsufflation manoeuvres each (Cough assist E70, Philips Respironics, Hamburg, Germany). Positive and negative pressures were set at the individual level of each subject.

### Measurements

Ventilation distribution was assessed by electrical impedance tomography (EIT) and multiple breath washout (MBW). Forced expiratory flows were measured by spirometry. These parameters were obtained five minutes before (baseline) and ten minutes after the treatment session (“post treatment”). Additionally EIT measurements were performed continuously during the treatment session.

#### Electrical impedance tomography

EIT is a non-invasive, radiation-free technique for the assessment of spatial and temporal ventilation distribution based on the changes in electrical properties of the tissue during the respiratory cycle. EIT measurements were performed using a commercially available setup (PulmoVista 500, Draeger, Germany). Image reconstruction was performed with the GREIT-algorithm using the torso mesh function^24^. Relative change in end-expiratory lung impedance (EELI) and a measure of ventilation inhomogeneity i.e. the global inhomogeneity (GI) index were calculated as described previously using customized software (Matlab R2013a, The MathWorks Inc., Nattick, MA, USA)^25–27^.

#### Multiple breath washout

All participants performed three trials of standard N_2_MBW until 1/40th of initial starting end-tidal N_2_ concentration according to the ERS consensus using an ultrasonic flowmeter equipment (Exhalyzer D, with Spiroware software, EcoMedics AG, Duernten, Switzerland)^28,29^. The resting time in between MBW trials was at least five minutes. The following parameters were calculated: lung clearance index (LCI), functional residual capacity (FRC_MBW_).

*Spirometry* was performed in three trials according to the ERS/ATS standards^30^ using the spirometer of a body plethysmograph (Jaeger Master-Screen Body/Diff device, CareFusion, Hoechberg, Germany). Forced expiratory volume in the 1st s (FEV_1_), forced vital capacity (FVC), peak expiratory flow (PEF), maximum expiratory flow at 50% vital capacity (MEF_50_) and forced mid-expiratory flow (FEF_25-75_) were assessed.

### Outcome measures

Primary outcome measures are the change in ventilation distribution assessed by N_2_MBW (LCI) and EIT (GI).

Secondary outcome measures include changes in forced expiratory flows and changes in end-expiratory lung volume from before to after the treatment session, breath by breath changes in EELI during the insufflation/exsufflation manoeuvre and the ratio of EIT amplitude (i.e. tidal volume) between insufflation/exsufflation manoeuvre and normal tidal breathing.

### Statistics

Given the lack of data on short-term effects on ventilation distribution, the sample size calculation was performed based on a study in subjects with cystic fibrosis investigating the effect of a physiotherapy treatment session ventilation inhomogeneity assessed by single breath washout^31^. The estimated sample size for a paired test was eight.

All analyses were performed in Statsdirect® Vers 3.3.3 (StatsDirect Ltd., Merseyside, UK). Data are presented as median and range. Changes in lung function parameters (primary and secondary outcomes) were assessed using Wilcoxon rank sum test. A *p*-value < 0.05 is considered significant. Breath by breath differences in EELI during the manoeuvre were analysed by single sample t-test assuming a mean difference of zero.

## Results

**Figure 1:**
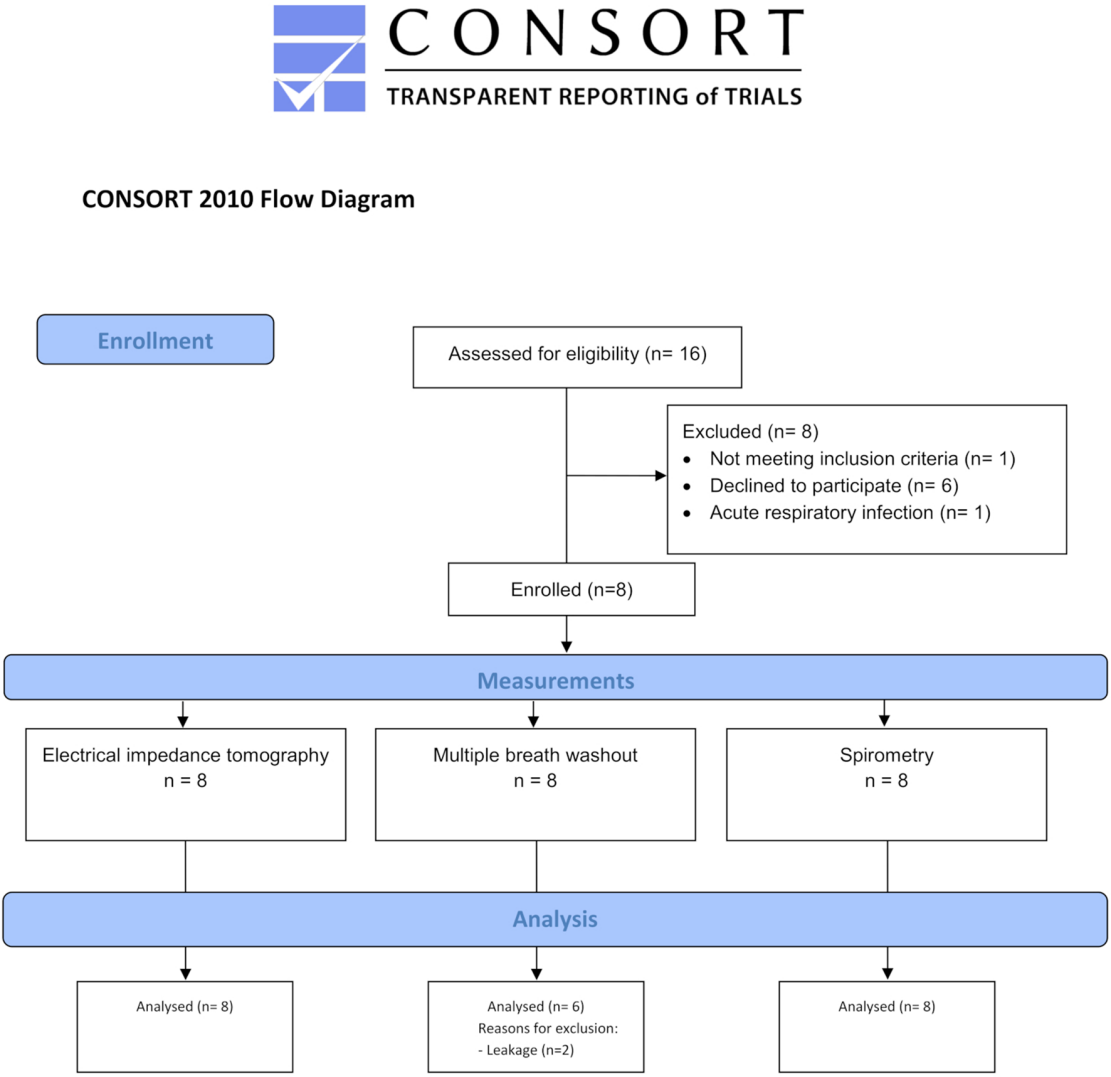
CONSORT Flow diagram.

Between April 2019 and November 2019 eight subjects (age 5.8–15.2 years) fulfilling the inclusion criteria could be recruited for the study (Fig. 1). Demographic data, diagnosis and individual pressure settings of the insufflator/exsufflator device are shown in Table 1.

**Table 1:** Demographic data including diagnosis, baseline vital capacity and device settings.

Age (years)	Sex	Diagnosis non-invasive ventilation	Weight(kg)	Height (cm)	FVC (l) [z-score]	Device settings
11.8	M	Nemaline myopathy *BiPAP during sleep*	34.4	134	0.49 [−7.00]	I: 25 mbar; 1.5 s E: −25 mbar; 1.5 s
12.3	F	Spinal muscular atrophy type II	26	144	0.84 [−2.23]	I: 25 mbar; 1.8 s E: −30 mbar; 1.5 s
10.1	M	Congenital muscular dystrophy SEPN 1 mutation	42.1	144	1.35 [−2.76]	I: 27 mbar; 1.7 s E: −30 mbar; 1.2 s
13.6	M	Spinal muscular Atrophy Type II *BiPAP during sleep (intermittent)*	28.8	141	0.37 [−7.87]	I: 30 mbar; 1.8 s E: −30 mbar. 1.5 s
15.2	M	Duchenne muscular dystrophy *BiPAP during sleep*	39.4	173	0.58 [−7.95]	I: 20 mbar; 1.0 s E: −25 mbar; 1.0 s
9.6	F	Congenital muscular dystrophy MDC1A Mutation	23.2	116	0.68 [−4.64]	I: 25 mbar; 1.2 s E: −31 mbar; 1.5 s
9.6	F	Spinal muscular atrophy type I C *BiPAP during sleep*	35.2	152	0.26 [−8.35]	I: 23 mbar; 1.3 s E: −25 mbar; 1.5 s
5.8	F	Spinal muscular Atrophy Type II	14.2	97	0.60 [−2.47]	I: 30 mbar; 1.0 s E: −35 mbar; 1.2 s

F: female; M: male; FVC: forced vital capacity; I: insufflation; E: exsufflation.

EIT measurements and spirometry could be performed in all subjects, technically acceptable MBW measurements could be achieved in 6 subjects. One subject with SMA Type 1c and one subject with Muscular dystrophy Duchenne failed MBW quality criteria for leakage due to insufficient seal around the mouthpiece.

**Figure 2:**
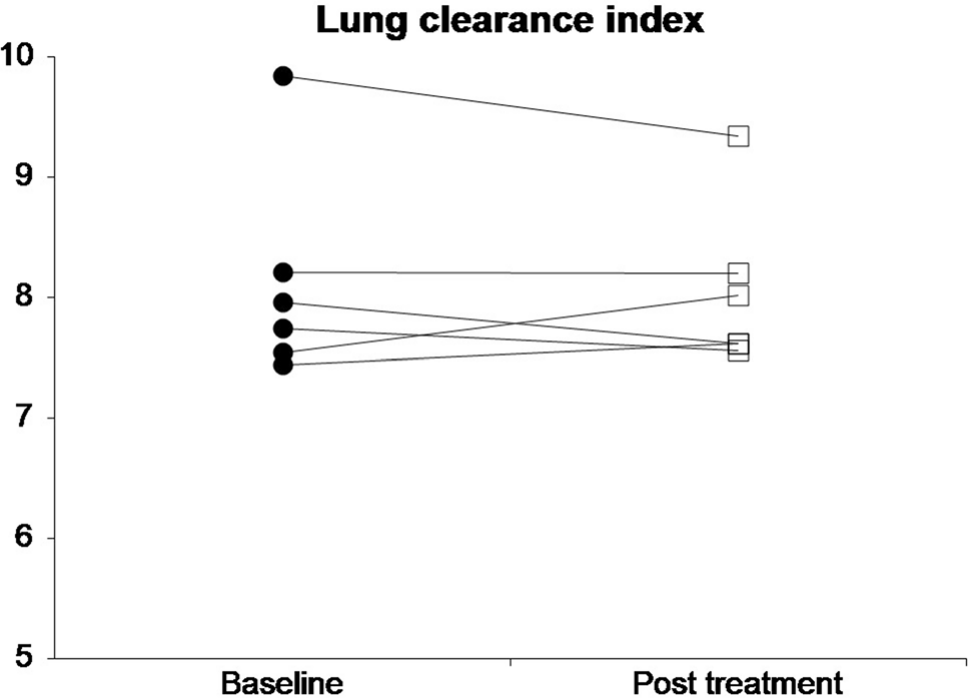
Individual changes in lung clearance index from baseline to post treatment.

**Figure 3:**
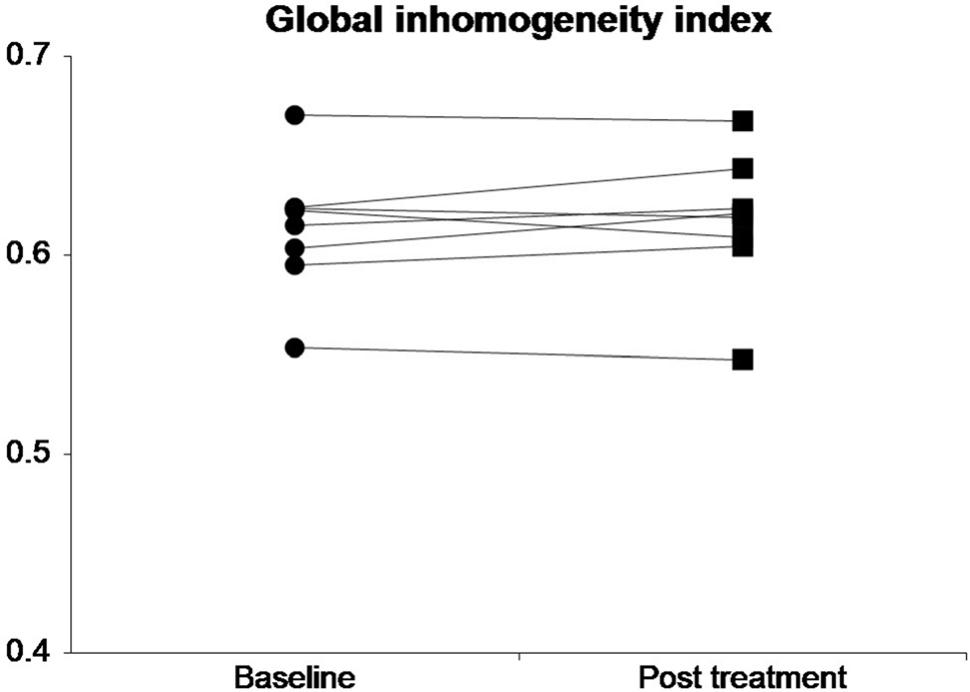
Individual changes in global inhomogeneity index from baseline to post treatment.

None of measured lung function parameters showed a significant difference between before and after an insufflation/exsufflation treatment session performed by a physiotherapist. All results are summarised in Table 2. The individual changes in the primary outcomes of each participant are shown in Fig. 2 and 3.

**Table 2:** Lung function tests before and after insufflation/exsufflation session results presented as median (range); *p*-value derived from Wilcoxon's signed ranks test.

	Baseline median (range)	Post treatment median (range)	Difference median (range)	*p*-value
LCI	7.85 (7.45–9.85)	7.82 (7.56–9.34)	0.09 (−0.47–0.51)	0.56
GI Index_EIT_	0.62 (0.55–0.67)	0.62 (0.55–0.67)	0.00 (−0.01–0.01)	0.46
FEV1 z-score	−5.78 (−7.03–−2.60)	−5.70 (−6.95–−2.52)	−0.06 (−0.15–0.12)	0.53
FVC z-score	−6.54 (−8.35–−2.47)	−6.38 (−8.29–−2.11)	−0.12 (−0.36–0.12)	0.25
PEF [litre/min]	1.39 (0.68–2.76)	1.51 (0.81–2.62)	0.32 (−0.44–1.55)	0.31
FEF_25-75_ z-score	−2.84 (−5.86–0.03)	−1.85 (−5.06–0.61)	0.32 (−0.44–1.44)	0.31
MEF_50_ z-score	−3.24 (−6.34–−1.36)	−2.30 (−6.50–−0.98)	−0.04 (−0.53–1.40)	0.64
FRC_MBW_ [litre]	0.99 (0.42–1.33)	1.03 (0.39–1.32)	−0.01 (−0.09–0.05)	0.58
ΔEELI_EIT_ [AU]			−0.16 (−0.35–1.11)	0.84

LCI: lung clearance index; GI Index_EIT_: global inhomogeneity index derived from electrical impedance tomography; FEV1: forced expiratory volume in 1 s; FVC: forced vital capacity; PEF: peak expiratory flow; FEF_25-75_: forced expiratory flow between 25 and 75% FVC; MEF_50_: mean expiratory flow; FRC_MBW_: functional residual capacity derived from multiple breath washout; ΔEELI_EIT_: difference in end-expiratory lung impedance normalised for tidal volume derived from electrical impedance tomography.

The breath amplitudes during the treatment were median 1.53 (range 1.43–2.05) times higher than during tidal breathing. The EELI during the insufflation/exsufflation manoeuvre did not differ on a breath-by-breath basis (*p* = 0.73).

## Discussion

In a small group of non-ambulatory subjects with neuromuscular disease, we found no changes in lung function parameters before and after a physiotherapy session with an insufflator/exsufflator device.

It is well known that subjects with NMD suffer from inspiratory and expiratory muscle weakness resulting in ventilatory failure, recurrent pulmonary infections and subsequently increased mortality^32–34^. Several studies suggest increased survival with the introduction of invasive and non-invasive ventilation^33^. Multiple other chest physiotherapy modalities are applied to improve airway clearance and to maintain vital capacity and effective cough^35,36^. Different studies investigating changes in lung volumes and cough efficacy show similar results. A small study by Katz et al.^37^ found that the decline in vital capacity was significantly attenuated, and assisted peak cough flow (PCF) was maintained in a clinically effective range. The same group followed 22 patients with DMD before and after the introduction of a lung volume recruitment technique (breath stacking). They demonstrated a significant reduction in loss of vital capacity over time after initiation of therapy^38^. Kang et al. observed an improvement of maximum inspiratory capacity in the majority of their patients trained to stacking delivered volumes of air to deep lung insufflation. Patients for whom the maximum inspiratory capacity increased also had a significant increase in assisted PCF despite having somewhat decreasing VCs and unassisted PCFs^39^. Stehling et al. performed a retrospective analysis of 21 patients with NMD. Before the introduction of mechanical insufflation/exsufflation vital capacity decreased from 0.71 ± 0.38 L to 0.50 ± 0.24 L in the last year and from 0.88 ± 0.45 L to 0.71 ± 0.38 L in the next to last year. In the first year, after regular use of mechanical insufflation/exsufflation vital capacity significantly increased by 28% (from 0.50 to 0.64 L). After the second year the vital capacity increase remained stable (0.64 vs. 0.65 L). The authors conclude that the regular use of mechanical insufflation/exsufflation might improve vital capacity in patients with neuromuscular disorders and severe lung volume restriction^3^.

The above presented studies all investigated mid- to long-term effects. Publications on short-term effects are very scarce. One study from Molgat-Seon et al. found an acute increase in respiratory system compliance immediately after lung volume recruitment (LVR) manoeuvres with a resuscitation bag in subjects with respiratory muscle weakness but not in controls. This effect could not be demonstrated any more one hour after the manoeuvre. LVR had no impact on lung volumes such as total lung capacity, functional residual capacity, inspiratory capacity and expiratory reserve volume. Peak expiratory flow was increased during LVR but not unassisted peak expiratory flow^40^. Another adult study by Meric et al.^23^ demonstrated positive effect on chest-wall motion asymmetry and a small but significant increase of vital capacity. The vital capacity return to baseline within one hour and the shallow breathing index increased significantly. Our data confirm the findings of Molgat-Seon et al. with a different LVR manoeuvre. Additionally, we can demonstrate that there is no short-term influence on ventilation distribution measured by LCI and also GI_EIT_. The difference of our findings to the latter study with respect to VC might be explained by the fact that an insufflation/exsufflation session can be tiring and potentially more so in children.

The main short-term aim of the insufflation/exsufflation technique is to improve cough and therefore expectoration of pulmonary secretions. Expiration is actively augmented with negative airway pressures. In doing so, airway clearance is enhanced eventually at the cost of the recruited lung volume achieved by the high inflation pressure. Therefore, short-term improvement of lung volumes and consecutively expiratory flows cannot be demonstrated in our investigation. We performed our measurement during a stable period without pulmonary exacerbations. The subjects are well trained in the application of the insufflation/exsufflation technique resulting in low amounts of secretion. The missing short-term effect of the therapy on ventilation distribution might indicate, that in these stable conditions neither secretions nor atelectasis are a major component of reversible ventilation inhomogeneity. Another explanation for our results might lie in negative pressures applied during expiration, leading to loss of a potentially recruited volume. Passive expiration after the last active insufflation or other techniques, such as air stacking might therefore be favourable in this respect. Insufflation/exsufflation manoeuvres can be tiring. This might be a further explanation for the lacking difference in VC, but not in ventilation homogeneity.

To our knowledge this is the first study evaluating not only lung volumes and expiratory flows but also measures of ventilation distribution to assess short-term effects LVR manoeuvres in subjects with NMD. Our lung function lab has a long standing experience and research interest in the applied lung function tests. All measurements hold the standard of ERS/ATS recommendations^28,41^.

Clearly a limitation of the presented study is the low number of subjects included. There are several reasons for this. First, for the sake of high quality lung function measurements, especially for ventilation distribution this work was designed as a single centre study. Second, to avoid heterogeneous results due to incorrect application of the insufflator/exsufflator, only subjects used to the therapy for several months were eligible for inclusion. One could argue that performing lung function measurements during a stable phase with respect to pulmonary symptoms is not the right time point to assess the therapy for short-term effects. This was a deliberate decision by the authors in order to gain insight into physiological conditions of these subjects. Furthermore, performing lung function tests during a pulmonary exacerbation is very stressful and not well tolerated at all by subjects with NMD.

## Conclusion

In a small group of stable paediatric subjects with NMD we could not demonstrate any short-term effects of insufflation/exsufflation manoeuvres on lung volumes, expiratory flows and ventilation distribution. This suggests that the beneficial effects of this technique is based on repetitive airway clearance and not so on immediate changes in lung function. A theoretical short-term benefit of augmented lung volume might be achieved by passive expiration after the last insufflation of the treatment session.

## Data Availability

The datasets analysed during the current study are available from the corresponding author on reasonable request.
